# Nanophotonic Image Sensors

**DOI:** 10.1002/smll.201600528

**Published:** 2016-05-30

**Authors:** Qin Chen, Xin Hu, Long Wen, Yan Yu, David R. S. Cumming

**Affiliations:** ^1^ Key Lab of Nanodevices and Applications‐CAS & Collaborative Innovation Center of Suzhou Nano Science and Technology Suzhou Institute of Nano‐Tech and Nano‐Bionics Chinese Academy of Sciences (CAS) Suzhou 215123 P. R. China; ^2^ School of Engineering University of Glasgow Glasgow G12 8LT UK

**Keywords:** image sensors, metamaterials, nanophotonics, plasmonics

## Abstract

The increasing miniaturization and resolution of image sensors bring challenges to conventional optical elements such as spectral filters and polarizers, the properties of which are determined mainly by the materials used, including dye polymers. Recent developments in spectral filtering and optical manipulating techniques based on nanophotonics have opened up the possibility of an alternative method to control light spectrally and spatially. By integrating these technologies into image sensors, it will become possible to achieve high compactness, improved process compatibility, robust stability and tunable functionality. In this Review, recent representative achievements on nanophotonic image sensors are presented and analyzed including image sensors with nanophotonic color filters and polarizers, metamaterial‐based THz image sensors, filter‐free nanowire image sensors and nanostructured‐based multispectral image sensors. This novel combination of cutting edge photonics research and well‐developed commercial products may not only lead to an important application of nanophotonics but also offer great potential for next generation image sensors beyond Moore's Law expectations.

## 1 Introduction

Digital image sensors (IS) that convert variable light intensities into electrical signals and constitute an image are important tool for the applications including photography, video imaging, condition monitoring and machine vision. The market size of complementary metal‐oxide‐semiconductor (CMOS) IS in 2014 reached 9 billion US dollars.^1^

The drive towards higher‐resolution imaging is ceaseless, resulting in a smaller sensor pixel. The state‐of‐the‐art commercial CMOS IS uses 1 μm × 1 μm pixels.^1^ Conventionally absorptive dyes are used as spectral filtering elements for color imaging by superimposing a dye‐doped polymer color filter on each pixel. The shrinking pixel size poses questions for color generation because of color crosstalk and carefully aligned lithography steps.^2^, ^3^ Recently, structured color based on the interaction between light and varying nanostructures rather than materials has attracted extensive interest because they create the possibility to overcome these issues and provide high compactness, desirable process compatibility, robust stability and tunable functionality.^4^, ^5^, ^6^ The impressive capability to print color at the diffraction limit with a resolution as high as 100,000 dpi shows promising potential for its applications in high‐resolution imaging and displays.^7^ In addition to the color filtering technique, other light manipulating requirements such as polarization and phased arrays based on nanophotonics have been proposed and demonstrated recently, with the potential for applications in image forming devices.^8^, ^9^, ^10^ This review will focus on the development and progress of ISs integrated with nanophotonic elements, in particular spectral filters. Since there are already several review articles on color filtering by nanophotonic techniques,^4^, ^11^, ^12^ this article will provide only a brief introduction to various nanophotonic color filtering techniques in Section 2. Typical parameters including transmittance and color purity will be summarized here. ISs using nanophotonic color filters made by various integration methods are reviewed in Section 3, where the size effect and the spatial crosstalk, considering the practical application in IS, are also discussed. Section 4 includes ISs based on alternative imaging techniques such as polarization imaging, multispectral imaging and THz imaging. Finally, Section 5 summarizes the progress of nanophotonic image sensors and discusses the future trends in the field.

## 2 Mechanisms of Nanophotonic Color Filtering

**Figure 1:**
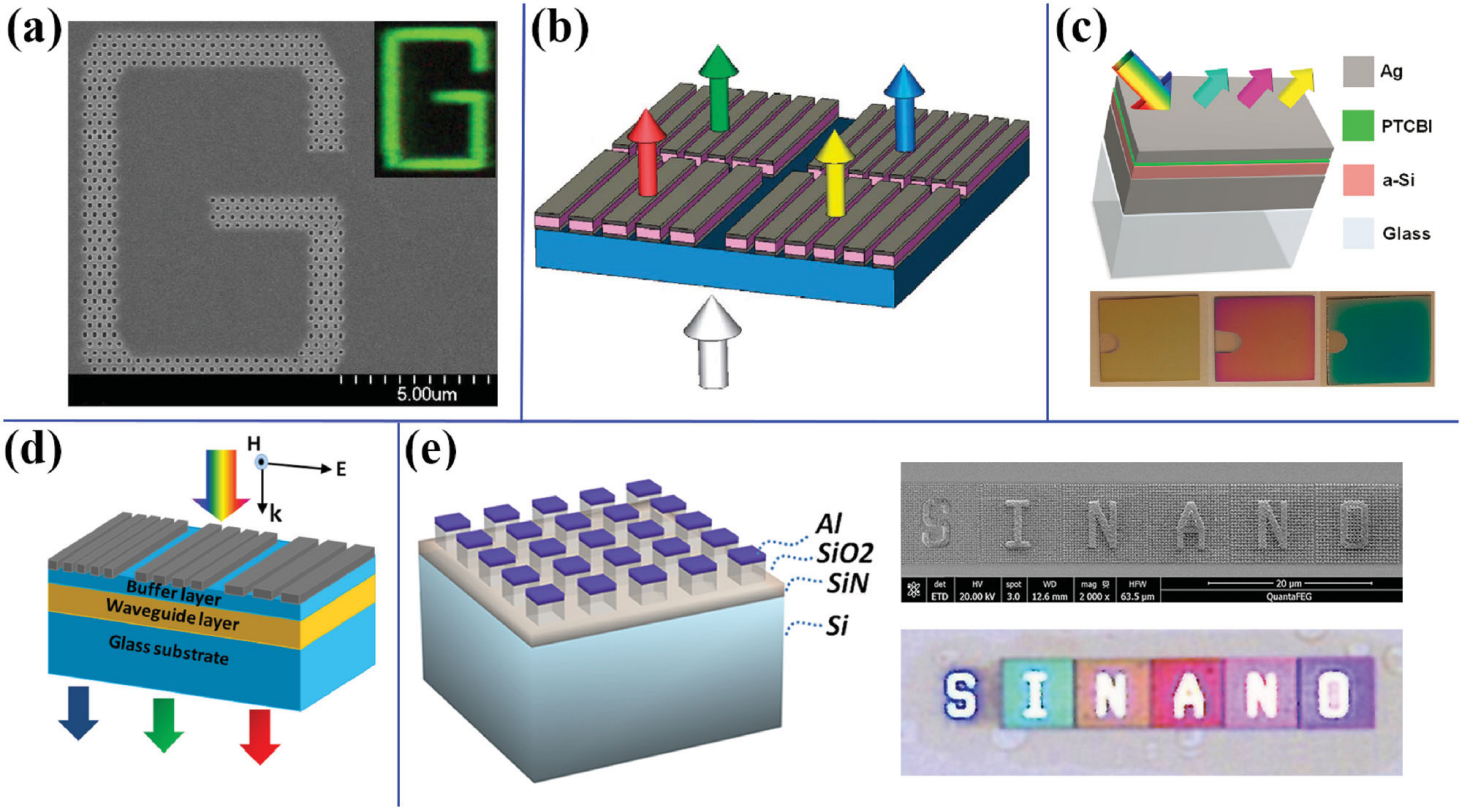
Various color filters based on nanostructures. a) Metallic nanohole array color filter. Circular holes are in a triangular lattice in 150 nm thick aluminium film, where the period is 330 nm and the diameter is 180 nm. A clear letter 'G' in green taken by microscope in transmission mode shows the great resolution down to about 1 μm. b) Metal‐dielectric‐metal grating color filters. The thickness of each layer in the stack is 40 nm (Al), 100 nm (ZnSe), and 40 nm (Al), respectively. The grating period is between 230–360 nm with a duty cycle of about 0.7. c) Planar cavity color filters. It is a reflective color filter based on Ag (18 nm)/PTCBI (5 nm)/a‐Si (14–34 nm)/Ag stack (180 nm). d) GMR color filters. The waveguide layer is made of Si_3_N_4_ with 100 nm‐thickness. The buffer layer is 50 nm thick SiO_2_ to suppress the metal absorption. The Ag gratings have a period of 250 nm to 450 nm with a duty cycle of about 0.75. e) Scattering color filters. It consists of 30 nm thick aluminium nanopatches on silicon with 80 nm thick SiO_2_ nanopatches as intermediate layer and 50 nm Si_3_N_4_ layer as antireflection coating. The period is 280–430 nm, and the sidelength is 100–220 nm. SEM and microscope color images of letters 'SINANO' are shown. a) Reproduced with permission.^16^ Copyright 2010, OSA. b) Reproduced with permission.^27^ Copyright 2010, NPG. c) Reproduced with permission.^36^ d) Reproduced with permission.^40^ Copyright 2011, AIP.

Structured color is based on the interaction between light and nanostructures. Various nanophotonic effects make it possible to selectively transmit or reflect light in a certain spectral band. Typical nanophotonic color filters are shown in **Figure** **1**.

i) Metallic nanohole array color filters as shown in Figure 1a are based on the extraordinary transmission (EOT) phenomenon.^13^ When a broadband light beam illuminates on the periodic nanohole array, only light in a pass band centered at the EOT wavelength could transmit through the array. Although the hole size is at a subwavelength scale, transmittance could be higher than the area filling ratio of the holes. By tuning the structure dimensions such as period and the material parameters, the EOT wavelength could be changed and thus the nanohole array acts as a transmissive color filter. Surface plasmonic resonance (SPR) is considered to play an important role in the color filtering, where incident light couples to surface plasmon resonance first and then couples back to light via periodic hole array for wave vector matching.^14^ Since the first paper by Ebbesen et al. in 1998, considerable efforts have been made to increase transmission, improve color purity, suppress angular sensitivity and improve design and fabrication efficiency.^15^, ^16^, ^17^, ^18^, ^19^, ^20^, ^21^, ^22^, ^23^, ^24^, ^25^, ^26^ Hole shape, lattice structure, index matching layer and multi‐period array were investigated both numerically and experimentally.
ii) The metal‐dielectric‐metal (MIM) grating color filters are based on the coupling between two metallic layers.^7^ Usually, there is at least one metallic layer patterned into nanostructures and the other metallic layer could have the same nanostructures, or complementary structures, or just be a continuous layer.^7^, ^27^, ^28^, ^29^, ^30^, ^31^ The gap plasmon resonance in this ultrathin stack shows wavelength sensitive spectral response. Not only the structure of each metal layer but also the distance between the two layers has significant effect on the spectral response. Such a structure can act as both transmissive and reflective filters and have more free degrees in design compared to metallic nanohole array color filters at a cost of relatively complex structures. In the example shown in Figure 1b, both metal layers and the dielectric layer were patterned into gratings, where the surface plasmon antisymmetric mode was used to design the color filters. The filtering colors can be tuned by varying the grating period. In addition, the filters are polarization sensitive, i.e. it only works for the traverse‐magnetic‐polarized waves (the electric field is perpendicular to the gratings).
iii) Planar cavity color filters have a major advantage over the above methods due to the lithography‐free fabrication. It could be as simple as a stack with one metal layer and a lossy semiconductor layer, where the perfect absorption induced by the critical coupling effect produces the color filtering phenomenon.^32^, ^33^, ^34^ Another top metal layer can be added to form a MIM stack as shown in Figure 1c, where the Fabry‐Perot (FP) cavity effect dominates.^35^, ^36^, ^37^, ^38^, ^39^ For a MIM stack, the intermediate layer do not have to be lossy but the lossy intermediate layer greatly reduces the conventional angular sensitivity of FP cavity filters. Note that no SPR is observed in this spectral filtering technique although it has the similar MIM stack as the MIM grating color filters. It could be transmissive filter if the metal is semi‐transparent or reflective filter if one thick metal layer acts as reflector. The filtering wavelength can be tuned by varying the thickness and refractive index of the intermediate layer.
iv) Guided mode resonance (GMR) filters consisting of diffraction gratings and a waveguide layer are widely used from visible to terahertz range.^40^, ^41^, ^42^ In some case, gratings with high refractive index could act as a waveguide layer simultaneously. The spectral filtering is caused by the selective coupling between the diffracted light and the waveguide mode following the wave vector matching condition. The matching condition is very sensitive to wavelength and incident angle. Therefore, it is applied as narrowband wavelength filter and demonstrates color tunability with the vision angle. Usually, dielectric and metallic gratings are used for reflective and transmissive filters, respectively.^43^, ^44^, ^45^, ^46^, ^47^, ^48^, ^49^ As shown in Figure 1d, red (R), green (G) and blue (R) filters can be obtained by tuning the metallic grating period. Varying the waveguide structure could also tune the filtering response. Multi‐band filters could be obtained by integrating multi‐period gratings or multiple mode waveguide.
v) Scattering color filters are based on the wavelength sensitive scattering of metallic particles.^50^, ^51^, ^52^, ^53^ Metallic nanoparticles support both light absorption and scattering. For the particles with a size larger than 100 nm, scattering dominates and is determined by the single particle material and profile. At the same time, the scattering was shown to be greatly modulated by the diffractive coupling between particles dependent on the interval and periodicity.^53^ SPR was found to greatly enhance the scattering from the particles. As shown in Figure 1e, aluminium nanopatch array induces strong backward scattering and shows distinct colors by tuning the size and the period. Because light is scattered preferentially into the optically dense medium, index matching layer of SiO_2_ is patterned below the metal patches. The SiN layer could reduce the reflection at the surface of silicon. Microscope color images of letters 'SINANO' generated from the aluminium patches can be seen.

**Figure 2:**
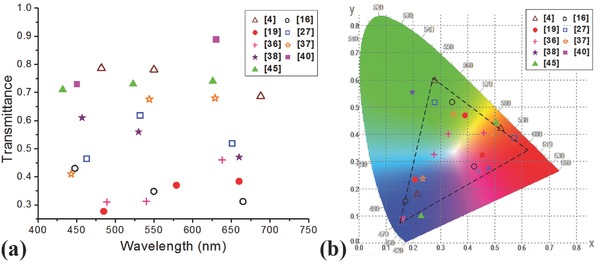
a) The peak transmittance and b) the passband width of various color filters. The black dashed lines in b) shows the color gamut.

In addition, there are some other mechanisms for color filtering including light diffraction of gratings, guided modes in nanowires and phase tuning.^54^, ^55^, ^56^, ^57^, ^58^, ^59^ No matter which techniques, light efficiency and color purity are two most important performance parameters for color filtering applications. Conventional dye based color filters absorb the unwanted light and transmit the desired light for color, where the transmittance is about 40% and increases to about 70% using microlens.^60^, ^61^ The passband width is about 100–150 nm. **Figure** **2**a shows the measured transmittances of various transmissive nanophotonic color filters and Figure 2b shows their chromaticity coordinates in CIE 1931 chromaticity diagram.^4^, ^16^, ^19^, ^27^, ^36^, ^37^, ^38^, ^40^, ^45^ As seen, the metallic nanohole array color filters have lower transmittances than the others, in contrast the GMR color filters have the highest transmittances. It is caused by the lossy surface plasmon resonance effect for the former and the dominated diffraction effect for the latter. The color purity is associated with the width of the passband. The narrow passband of GMR filters and planar cavity MIM filters ensure high color purity.^38^, ^40^ However, planar cavity MIM filters with a lossy intermediate layer has low transmittances and poor color purity.^36^ Although GMR filters have both high transmittances and color purity, the angular sensitivity may hamper their applications in imaging.^49^ Metallic nanohole array color filters is easy to be used as electrodes and therefore may be integrated into active devices to make it tunable or multifunctional.^46^ Considering the application in ISs, the integration is another criterion. The planar cavity color filters are easy to fabricate in large scale that fits for display application. But for high resolution imaging, the required aligned multistep lithography is still difficult when the pixel size is down to 1 μm. Therefore, there are still a lot to do to improve various nanophotonic filtering techniques and optimizations are requested based on applications.

## 3 IS with Nanophotonic Color Filters

**Figure 3:**
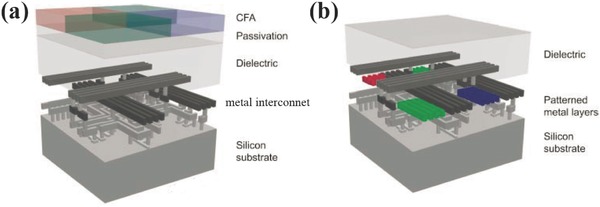
a) Schematic of conventional IS with the dye color filter array on top of the sensor. b) Schematic of plasmonic IS with the optical elements integrated in the Metal layers. Reproduced with permission of the Copyright holder, Ricardo Motta.^63^

A regular IS has a typical structure as shown in **Figure** **3**a, where silicon is the active material for photosensing, metal layers separated by dielectric layers are for electric interconnection, color filter array (CFA) is integrated on top. This configuration is called front‐side illumination (FSI), i.e. the light is coming from the front side of the chip where the CFA is positioned.^1^ Usually the CFA is about 1 micron thick and it needs aligned multistep processing for R, G, B filters. With the increasing of the imaging resolution, i.e. the decreasing of the pixel size, the fabrication becomes challenging and the spatial color crosstalk gets serious.^2^, ^3^ Back‐side illumination configuration is an alternative choice to resolve these issues by etching the backside of the silicon substrate and then integrating the CFA on the back side, but it is relatively complex. In 2001, Catrysse and Wandell proposed the concept of integrating nanophotonic components inside the IS chips based on the existing metal interconnect layers.^62^ As shown in Figure 3b, the metal layers could be patterned into various nanostructures as those shown in Figure 1, and thus they could behav e as color filters.^63^ Compared to the top surface integrated dye filters, the metallic ones is ultrathin (≈100 nm), heat and radiation robust, and it has spectral tunability, easy to be arrayed (single step patterning) and low crosstalk due to the reduced vertical distance to the photodiodes.^64^, ^65^, ^66^ Furthermore, near field SPR enhancement of metallic nanostructures could improve the photoelectrical response.^67^, ^68^ As an extension, nanostructure based lens and polarizers could be integrated as well, and not only the metallic optical components but also the dielectric ones could be used.^49^, ^54^, ^69^, ^70^, ^71^ In this section, recent development and progress of IS integrated with nanophotonic color filters are reviewed. They are sorted into three types, 1) IS with attached filters that are fabricated on other carriers,^72^ 2) IS with filters integrated on top via post‐CMOS process,^73^, ^74^, ^75^ 3) IS with filters integrated via the back‐end‐of‐line CMOS process.^76^, ^77^, ^78^

### 3.1 Size Effect of Nanophotonic Color Filters

**Figure 4:**
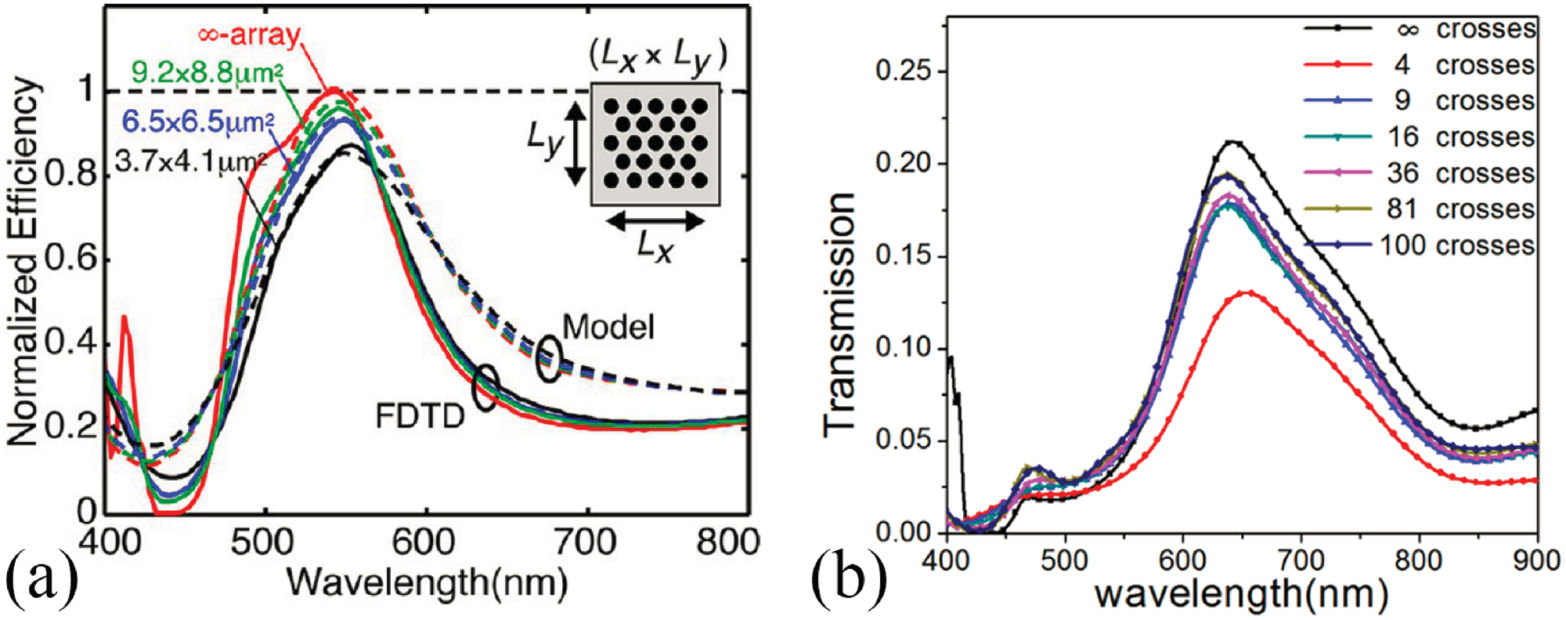
a) Normalized transmission spectra of nanohole array in 150 nm thick aluminium film. The period is 340 nm and the diameter is 180 nm. b) Transmission spectra of nanocross array in 200 nm thick aluminium film. Period is 280 nm, sidelengths of the arms of the cross are 200 nm and 50 nm. 4, 9, 16, 36, 81 and 100 nanocrosses occupy 0.6 × 0.6, 0.89 × 0.89, 1.18 × 1.18, 1.8 × 1.8, 2.66 × 2.66, 2.96 × 2.96 μm^2^. a) Reproduced with permission.^72^ Copyright 2013, ACS. b) Reproduced with permission.^80^ Copyright 2015, OSA.

As shown in Figure 1, most types of nanophotonic color filters rely on the nanostructure array. Usually the transmissive or reflective spectra are simulated with periodic boundary condition and the measured spectra are from a sample with large patterned area. However, in practical applications, the size of the color filter is limited to that of the sensor pixel, which is down to 1 μm for the state‐of‐the‐art devices.^1^ In this case, it is necessary to find out the exact filtering performances of nanophotonic color filters in such a limited pixel area. There are some works on the size effect of the color filters.^72^, ^79^, ^80^ As shown in **Figure** **4**a, the transmittance decreases with the decreasing area of the circular hole array. About 20% reduction was observed for a nanohole array color filter with a size of 3.7 × 4.1 μm^2^. For even smaller filter sizes, the similar trend was observed as shown in Figure 4b, where the transmittance drops by a half at a size of 0.6 × 0.6 μm^2^. On the other hand, the peak transmission wavelength has little shift, i.e. the displaying color of the filter is less sensitive to the filter size as also demonstrated in Reference 7. The data in Figure 4 are all for metallic nanohole array color filters, but the property is common to other nanostructured ones, which needs to be considered in the image sensor applications.

### 3.2 Spatial Color Crosstalk in Nanophotonic IS

**Figure 5:**
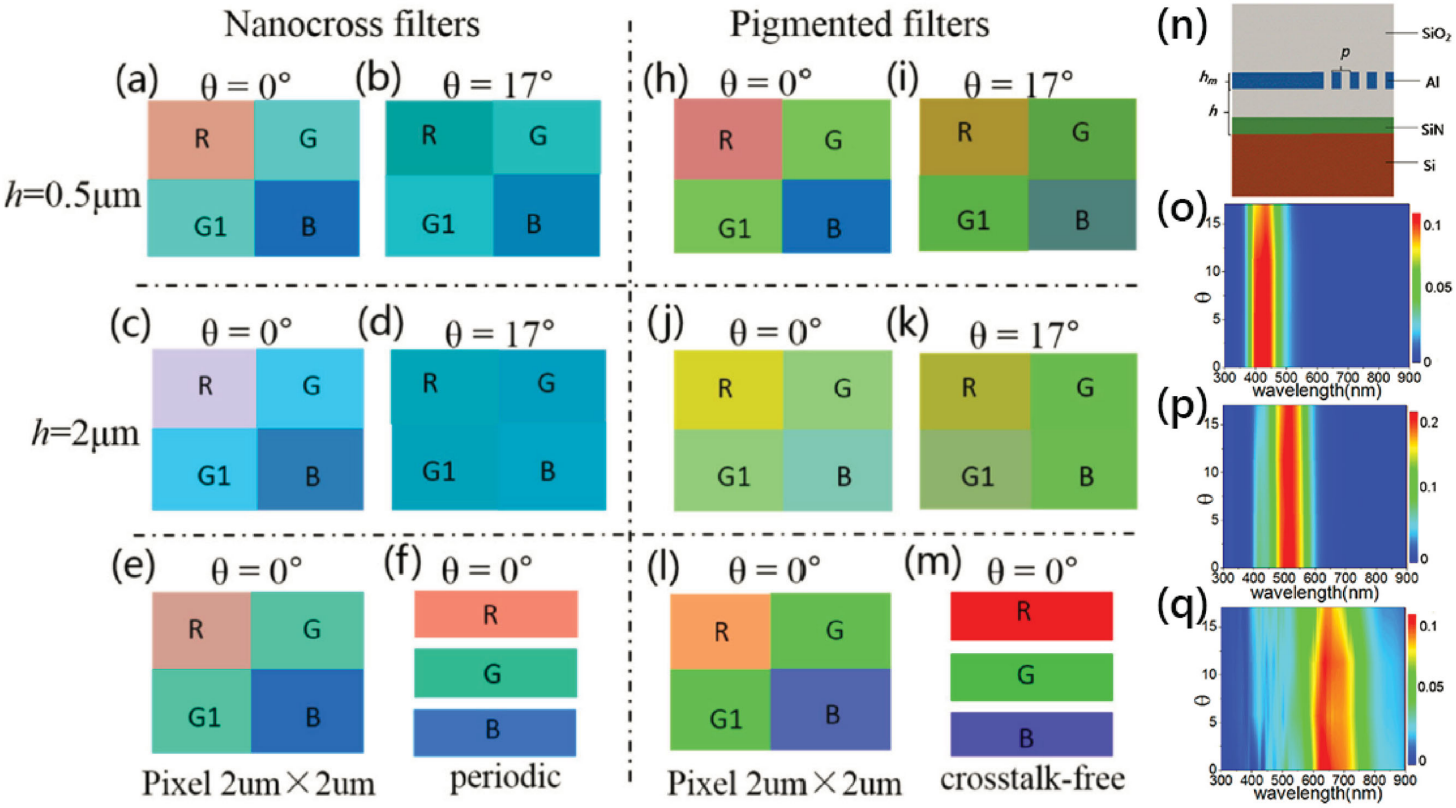
a) The calculated colors of R, G and B pixels in IS using a–f) nanocross filters and h–m) dye filters. n) is the simulation model of IS. o–q) are the transmission spectra of three filters versus illumination angles. a)–d) and h)–k) are for 1 μm × 1 μm pixels. e) and l) are for 2 μm × 2 μm pixels. a), b), h) and i) are at *h* = 0.5 μm. c)–e) and j)–l) are at *h* = 2 μm. f) and m) are the reference colors of periodic nanocrosses filters and individual pigmented filters regardless of crosstalk. a–q) Reproduced with permission.^80^ Copyright 2015, OSA.

Color crosstalk turns out to be a serious issue of IS with the pixel size going down to sub‐2 μm.^3^, ^60^, ^61^ There are spectral crosstalk, spatial crosstalk and electrical crosstalk, where the former two are related to the color filter design. Usually, spectral crosstalk is raised due to the wide passband of filters, and the spatial crosstalk is related to the filter position, the illumination angle and the pixel size. Obviously, the spatial crosstalk is the major optical factor of the crosstalk for high resolution imaging. It is expected that integration of nanophotonic color filters inside the IS chip as shown in Figure 3b could greatly reduce the spatial crosstalk due to the decreasing lateral shift of the light beam in a small vertical distance. As shown in **Figure** **5**, the spatial color crosstalk of IS with nanocross array filters were investigated quantitatively.^80^ Nanocross array filters used have less angular sensitivities as shown in Figure 5o–q, which has negligible effect on the color distortion. Compared to the original colors in Figure 5f, obvious color distortions were observed at a pixel size of 1 μm × 1 μm when the filters were placed at a distance *h* = 2 μm away from the photodiodes as shown in Figure 5c, which is even worse for oblique incidence in Figure 5d. The issue was found to be suppressed by placing the filters close to the photodiodes, for example *h* = 0.5 μm as shown in Figure 5a and 5b. The dye filters also show significant color crosstalk, which in principle could be reduced similarly. However, the easy integration of the nanocross filters to the existing Metal connection layers that close to the photodiodes may fit better for the standard IS processes.

### 3.3 IS with Attached Nanophotonic Color Filters

**Figure 6:**
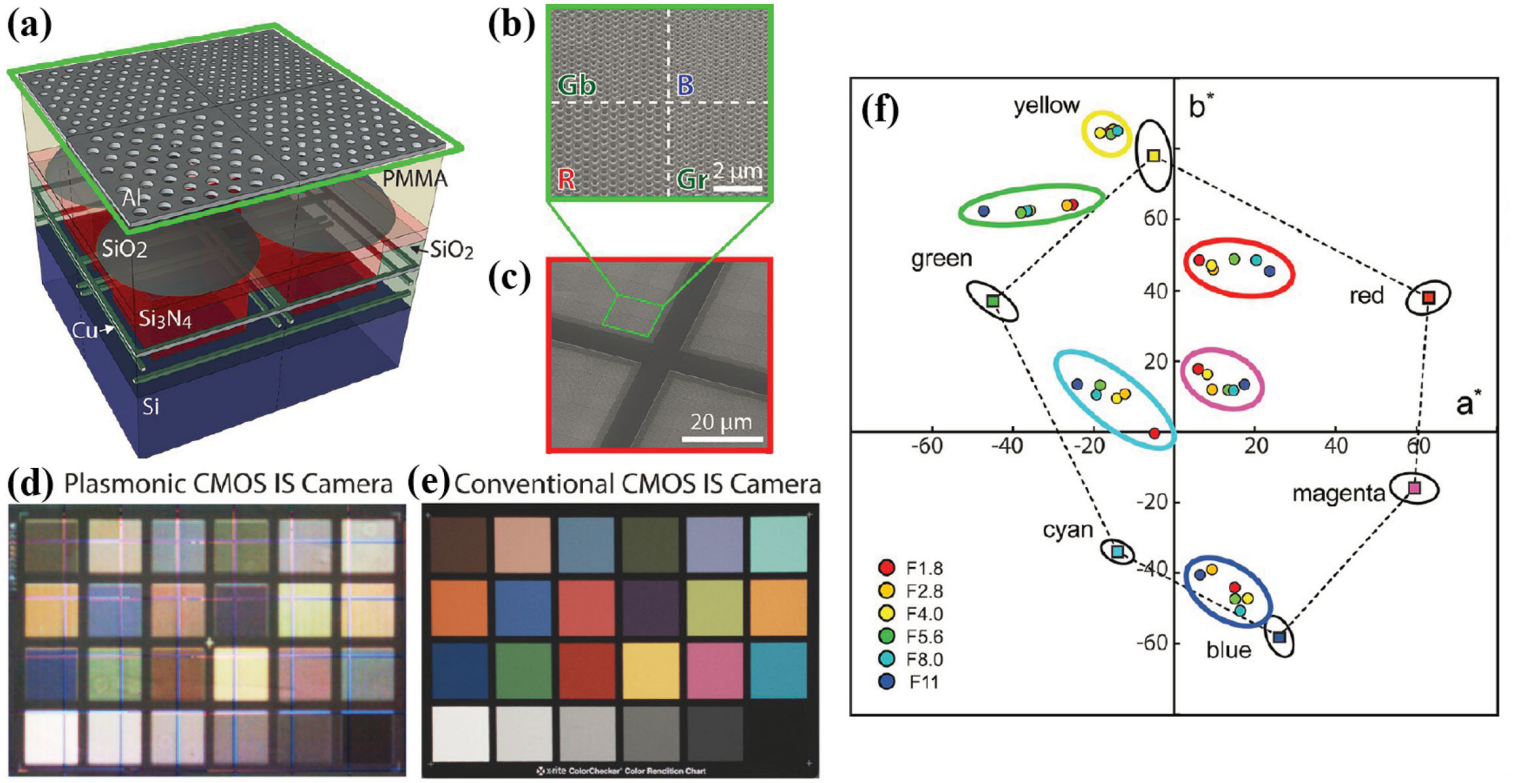
a) Nanohole array color filter attached onto IS. b,c) SEM images of nanohole array. d,e) Images of a 24‐patch Macbeth color chart taken with the plasmonic IS camera and the conventional IS camera, respectively. f) CIE L*a*b* chromaticity diagram for the performance of the integrated hole array color filter IS as a function of f‐number. a–f) Reproduced with permission.^72^ Copyright 2013, ACS.

For IS application, transmissive color filters are preferred. So far, most transmissive nanophotonic color filters were fabricated on glass for easy characterization.^13^, ^14^, ^15^, ^16^, ^17^, ^18^, ^19^, ^20^, ^21^, ^22^, ^23^ The simplest way to test the functionality of the nanophotonic color filters for ISs is to place the CFA on glass directly on top of a black and white image sensor pixel array with PMMA as the intermediate layer, proposed and demonstrated by Atwater et al. in 2013.^72^ As shown in **Figure** **6**a, the used ISs are commercial products with microlens array, and the color filters consist of silica filled hole array in aluminium film on glass. Each filter has a size of 5.6 × 5.6 μm^2^, which is twice that of the pixel, to account for alignment errors in the integration. Both the simulation and the measured response confirmed a high coupling efficiency by this simple physical contact integration. By measuring color difference Δ*E* between the reference color on a Macbeth color chart and what the color actually is in the signal‐processed image as shown in Figure 6d and 6e, it was found that the nanohole array color filter is efficient for short wavelength (blue) but has large spectral crosstalk for long wavelength (green and red). The spectral crosstalk is indicated by the distance between the color circles (integrated color filters) and the color squares (standard colors) as shown in Figure 6f. The dependence of the filtering quality on the f‐number was also investigated. The results show that these nanohole array color filters are quite angular robust. The spectrum taken with a lens (f‐number = 1.8, 15° acceptance angle) is similar to the normal incidence one.

### 3.4 IS with Post‐CMOS Processed Nanophotonic Color Filters

**Figure 7:**
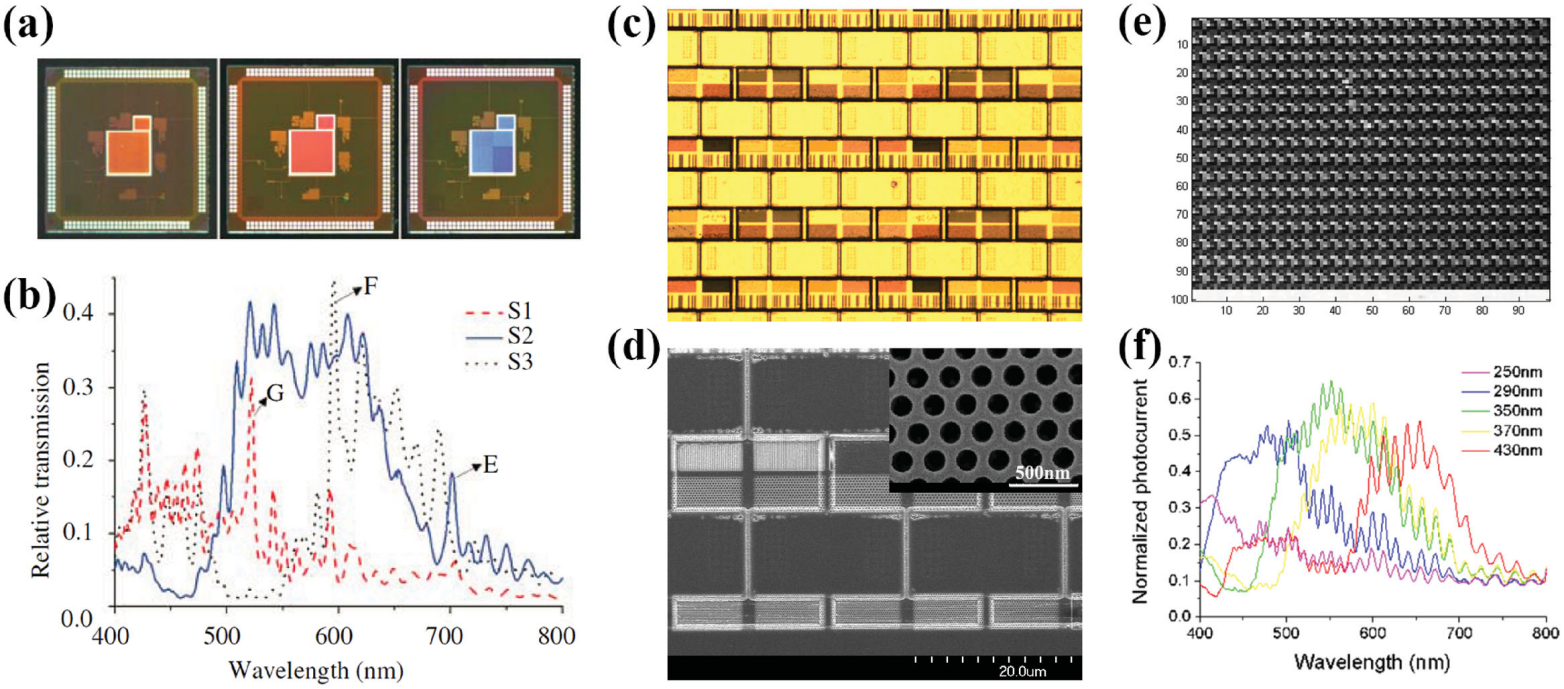
a) Microscope images of single pixel ISs with different electron beam lithography patterned nanohole array filters. b) Photocurrents of the ISs in (a) normalized to the bare IS. c) Microscope image of IS integrated with nanohole array color filters. d) SEM image of pixel array with nanohole array filters. e) Photocurrent distributions across the 100 × 100‐pixel photodiode array. f) Photocurrents of photodiodes in c) with integrated filters normalized to the bare one. a, b) Reproduced with permission.^74^ Copyright 2012, IEEE. c–f) Reproduced with permission.^75^ Copyright 2012, Springer.

Other than attaching filters on glass to ISs, the nanophotonic color filters can be also directly fabricated on ISs via the post‐CMOS process, which provides a better way to align the filter array to the photodiode array but does not have to access the back‐end‐of‐line process in CMOS foundry. In 2008, Swiss Center for Eelectronics and Microtechnology (CSEM) investigated the possibility to fabricate plasmonic nanostructures and multilayer replication grids as spectral filters to replace the printed color filters in their surface modified vision systems.^73^ Well separated spectral response from red to near infrared were demonstrated by the plasmonic filters with a passband of about 70 nm. In 2012, Qin and Cumming et al. demonstrated nanohole array color filters integrated ISs, where the nanophotonic filters were fabricated using electron beam lithography on top of the passivation layer.^74^, ^75^ In this way, the alignment between the filter array and the pixel array could be improved compared to that in Section 3.3. The photocurrent distribution in **Figure** **7**e shows pretty good uniformity across the whole 100 × 100 pixel array. The pixel size is about 4.5 μm × 9 μm, resulting in a good filtering response of the nanohole filters. Although the interference of light reflection at all interfaces induces fluctuations in the measured photocurrents spectra, the distinct spectral filtering responses of the different filters still can be observed as shown in Figure 7b and 7f. In fact, an ideal way is to etch away the dielectric passivation layers on top of photodiode and then fabricate nanophotonic color filter at the position close to the silicon diode, in which case SPR of the metallic nanostructures could further improve the photosensitivity.^67^, ^68^ However, the SiO_2_ and Si_3_N_4_ passivation layers grown in semiconductor foundry are quite thick and etch‐resistive. For example, four and six Metal layers were used for the chips in Figure 7a and 7c, which means several microns thick passivation layers. So it is pretty hard for lab fabrication.

### 3.5 IS with Back‐End‐of‐Line CMOS Processed Nanophotonic Color Filters

Both applications of nanophotonic color filters in IS in Section 3.3 and 3.4 demonstrate the expected functionality. But in practice, completely integration of color filters by back‐end‐of‐line CMOS process is preferred. It is not easy to access for academia. Fortunately, industry players contribute a lot in this direction.

**Figure 8:**
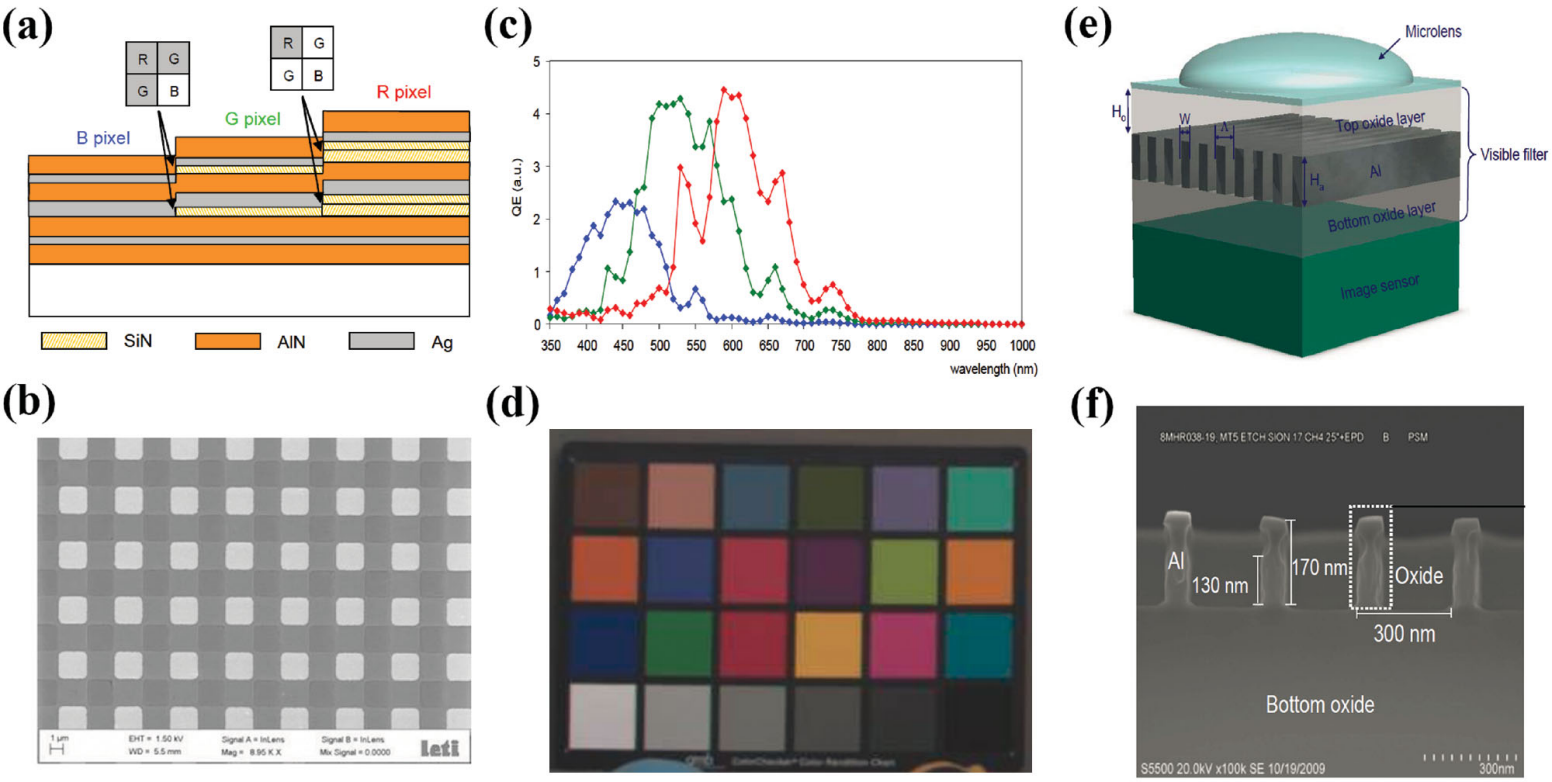
Planar cavity color filters integrated IS. a) Schematic of the MIM planar cavity color filters. b) SEM image of the pixel array. c) Measured quantum efficiency spectra without microlens. d) A color image recorded by this IS. e) Schematic of aluminium grating color filter integrated IS. f) SEM image of the grating color filter. a–c) Reproduced with permission.^77^ Copyright 2011, OSA. d) Reproduced with permission of the Copyright holder, Laurent Frey. e,f) Reproduced with permission.^76^ Copyright 2010, IEEE.

In 2011 CEA LETI and ST Microelectronics demonstrated an IS with planar cavity color filters similar to the one discussed in Section 2.^77^ As shown in **Figure** **8**a, a 7‐layer stack with two thickness tunable layers was used to realize the R, G, B colors, which requires four photolithography levels. The SEM image in Figure 8b shows the good fabrication quality of the array (pixel size 1.75 × 1.75 μm^2^), where the misalignment between four levels was estimated to be around 200–250 nm. The quantum efficiency was measured from large uniform stripes where neighbouring pixels has the same filter color, i.e. no color crosstalk was included. As shown in Figure 8c, R, G, B responses were clearly distinct. The oscillations were caused by the interference as that in Figure 7. Due to the total thickness of these filters are only a half of the traditional dye filters, the color crosstalk in the practical Bayer array was expected to be reduced. A 3Mpixel color image was taken using this IS and showed nice image quality. However, 200–250 nm misalignment of the four lithography level process of the planar cavity color filters would be a serious issue for IS with the pixel down to 1 μm. In addition, silver was used in the color filter stack but it is not a standard CMOS compatible metal material.

Yoon and Lee with support from Siliconfile Technologies reported one dimensional grating color filters integrated in IS.^76^ Using a 90 nm CMOS process, aluminium grating color filters with a width of 90 nm and a period of 260–360 nm were fabricated. The subwavelength metallic gratings act as low‐pass wavelength filters for transverse electric (TE) polarized light, where the peak transmittance as high as 80% was obtained. The advantage of these filters for integration is that various color filters could be patterned in one‐step lithography by just tuning the period for different pixels. Unfortunately the low‐pass filters have poor color purity and show a polarization sensitivity.

**Figure 9:**
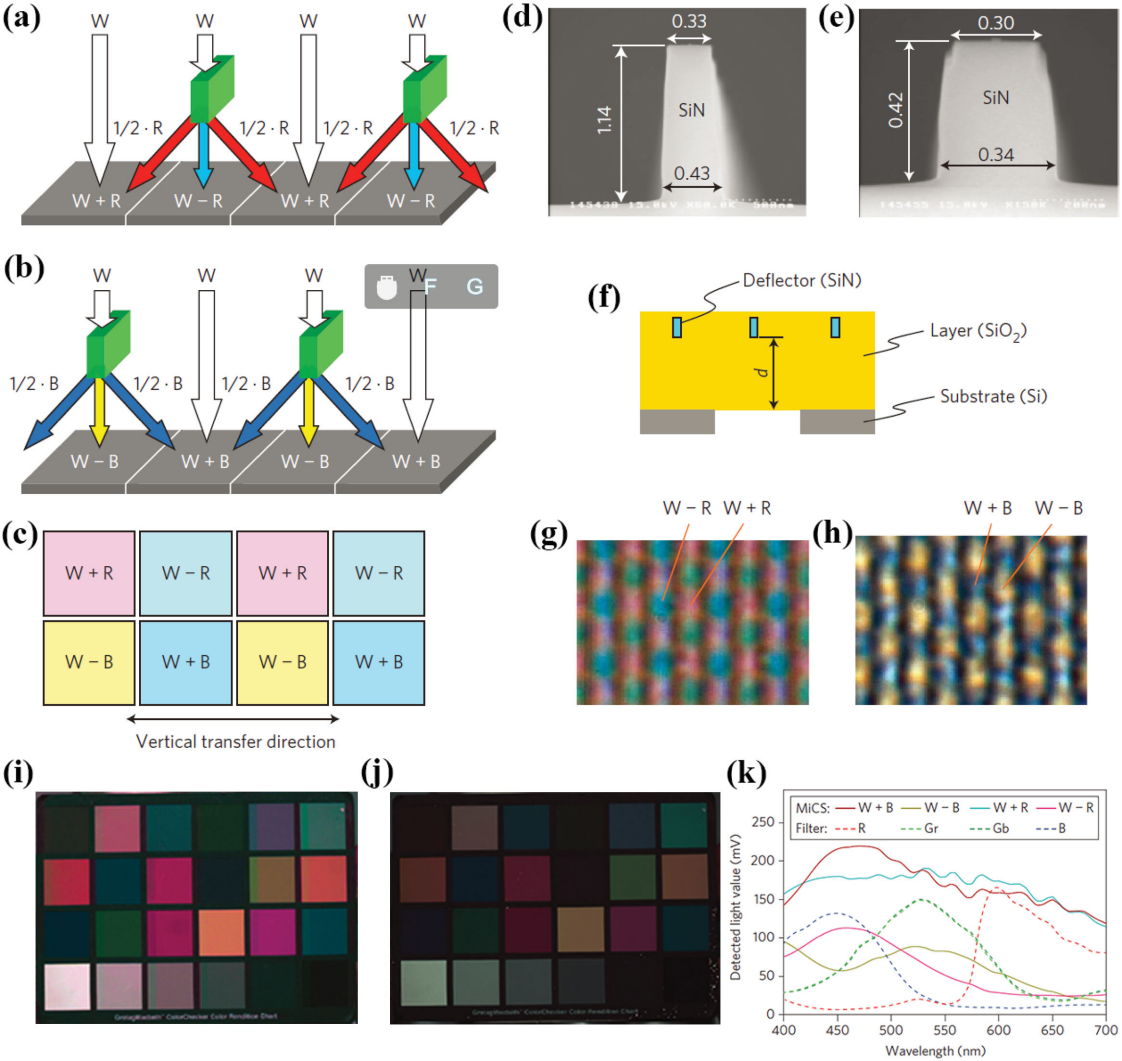
Dielectric color splitters integrated IS. a,b) Schematic of the R and B splitters. c) The color splitter array for imaging. d,e) SEM images of the R and B splitters. f) Cross‐sectional configuration diagram of the evaluated sample. d = 4.97 and 5.02 μm for R and B splitters. g,h) Microscope images for the transmitted light of the R and B splitter array. i,j) Photographic images from the proposal IS and the conventional IS. k) Experimental spectroscopic characteristics of the proposed IS. Reproduced with permission.^78^ Copyright 2010, NPG.

Panasonic Corp. proposed in 2010 a color splitting method for color imaging in IS.^78^ As shown in **Figure** **9**a and 9b, dielectric plate‐like structures were used to deflect light, which is determined by the wavelength sensitive phase difference between the light propagating through the deflector and that through the surrounding medium. Combining the R and B splitters, four colors W+R, W–R, W–B and W+B (W means white) can be obtained for color imaging. The SEM images of the splitters (0.3 μm in width) and the microscope images of the splitter array (1.43 μm pixel) show the potential for high‐resolution imaging. As expected, the color image of the proposed IS is brighter than that of the conventional IS with dye filters. Quantitatively, the amount of light received by the detector is 1.85 times higher. However, the color purity of the color splitting technique is poor as shown in Figure 9k and the color distortion can also be seen from Figure 9i.

From recent progress in ISs with nanophotonic color filters, promising potential has been demonstrated no matter which color filtering technique or integration method. The spectral engineering capability and the chip integration compatibility of nanostructures make it possible for next generation ISs with better color purity in a higher resolution.

## 4 Other Nanophotonic ISs

Although color imaging, as discussed in Section 3, is the most widely used and mature imaging technique, spectral and functional extensions to this technique are very important to security, bioresearch, and aerospace technology. In this Section, filter‐free nanowire IS, polarization imaging IS, terahertz imaging IS and multispectral IS based on nanophotonic techniques are reviewed.

### 4.1 Nanowire Based Filter‐Free IS

**Figure 10:**
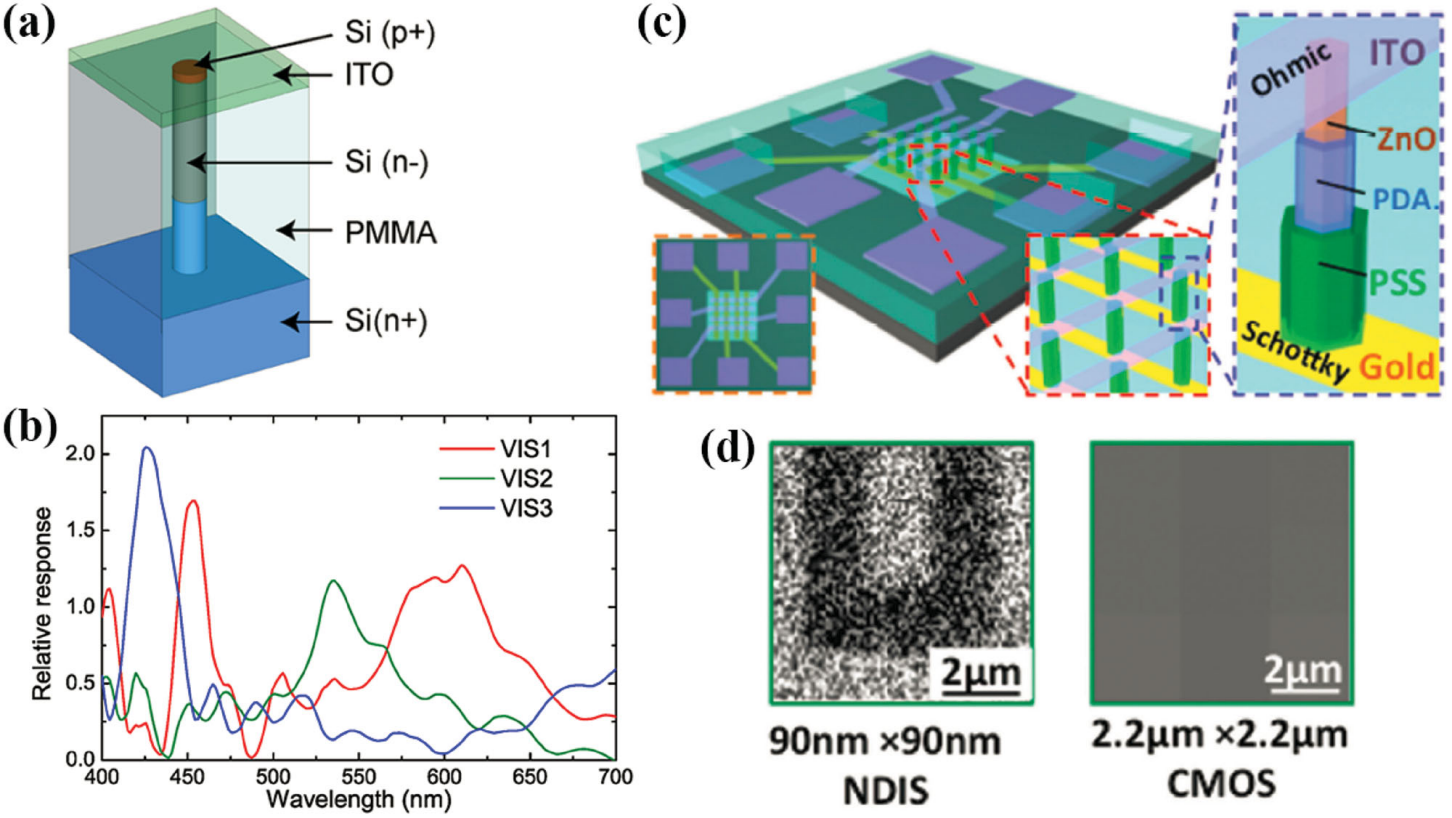
a) Schematic of the silicon nanowire imager. b) Measured spectral response mimicking the CIE 1964 XYZ color matching functions. c) Schematic of the ZnO nanowire imager. d) The projection images recorded by the NDIS and the conventional IS. a,b) Reproduced with permission.^81^ Copyright 2014, ASC. c,d) Reproduced with permission.^82^

As mentioned in Section 2, silicon nanowires have been used as color filters based on light guiding in individual nanowire.^57^, ^58^, ^59^ Since silicon is photosensitive, silicon nanowire could be used as color selective photodetector, or named filter‐free IS.^81^, ^82^ Compared to the ones in Section 3, this type of IS could be ultracompact and high efficiency. In 2014, Crozier et al. demonstrated such a IS as shown in **Figure** **10**a. Vertically doped silicon nanowire is surrounded by PMMA and acts as wavelength sensitive photodetector by itself, where the wavelength is tuned by the nanowire diameter.^81^ The measured results from the nanowire pixels show distinct spectral response mimicking the standard color matching functions as shown in Figure 10b. Due to the large component around 450 nm for the VIS1 pixel that supposed to be R filter, the images taken by this IS shows color distortion to blue. Similarly, Song et al. presented ZnO nanowires IS to improve the image resolution in 2015, where the nanorod digital image sensor (NDIS) has a pixel as small as 50 × 50 nm.^82^ As shown in Figure 10d, the image recorded by NDIS clearly shows U‐shape pattern but nothing useful was recorded by the conventional IS.

### 4.2 Polarization IS

**Figure 11:**
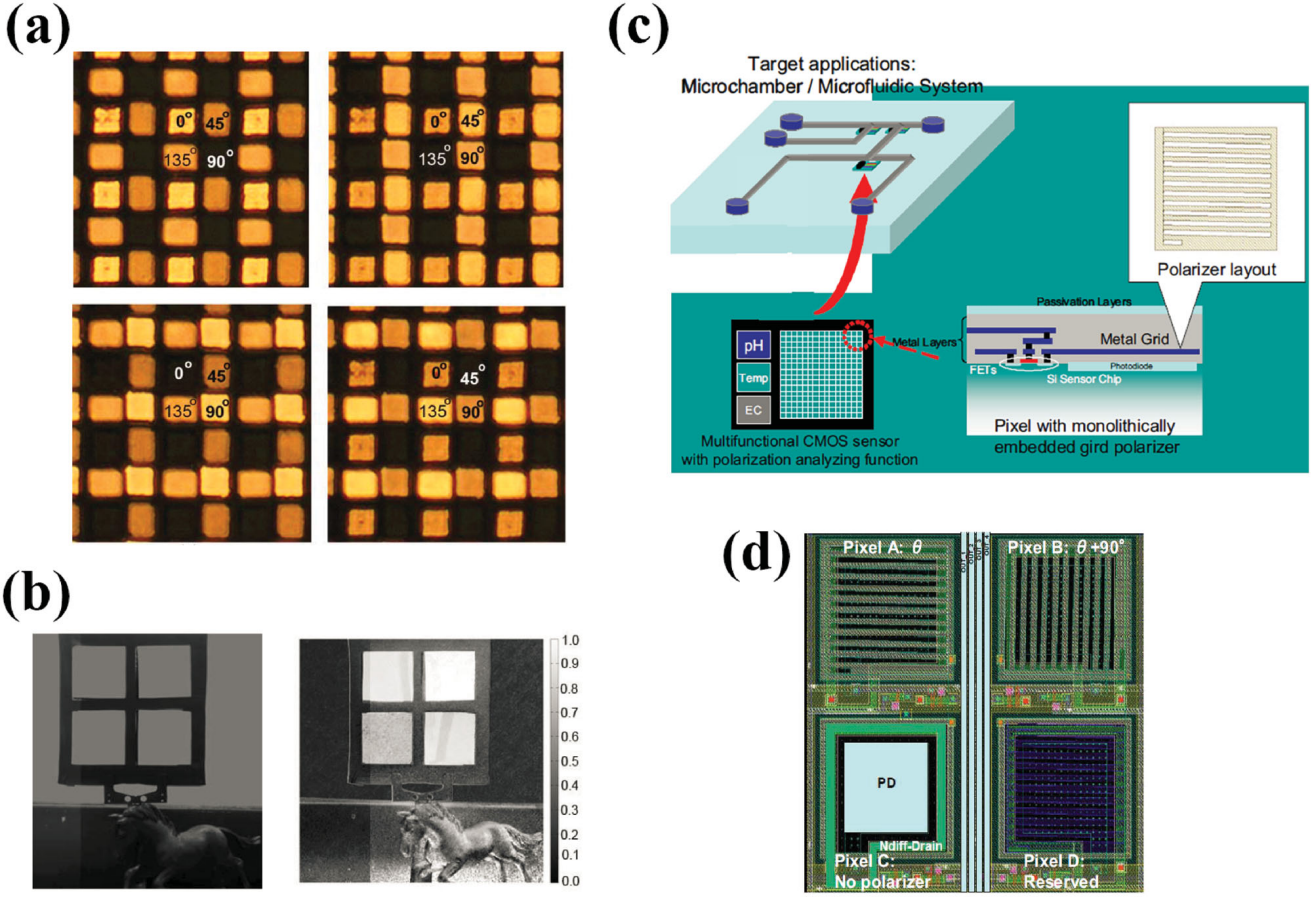
a) Microscope images of the micropolarization pixel array illuminated with 0°, 45°, 90°, 135° polarized incident light. b) Sample images obtained from regular CCD image sensor (left) and polarization CCD image sensor (right). c, d) Schematics of the microfluidic system integrated polarization IS and the wire grid polarizer integrated pixels. a, b) Reproduced with permission.^88^ Copyright 2010, IEEE. c, d) Reproduced with permission.^89^ Copyright 2008, IEEE.

To measure a polarization image, multiple images of the same scene are required for different orientations of the linear polarizing filter, which is usually realized by placing a linear polarizer in front of a regular image sensor.^83^, ^84^ It is bulky, slow and difficult to use. Micropolarizer array was proposed to integrate the polarization image function down to pixels, in which case the polarization image can be taken as simple as color image sensors. Compared to iodide‐doped polyvinyl alcohol (PVA) films or liquid crystal micropolarizers,^85^, ^86^ metallic wire grid polarizers have similar advantages of nanophotonic color filters over dye color filters.^87^ As shown in **Figure** **11**a, the wire grid polarizers were patterned on a charge‐coupled device (CCD) IS along different orientations into a Bayer array.^88^

The microscope images clearly show the dependence on the illumination polarization. The polarization image taken by the IS shows improved details compared to the regular IS, indicating the polarization imaging function of the wire grids. Similarly, the metallic wire grid polarizer integrated IS was packaged with microfluidic system for polarimetric measurement of the chiral solutions as shown in Figure 11c.^89^

### 4.3 THz IS

**Figure 12:**
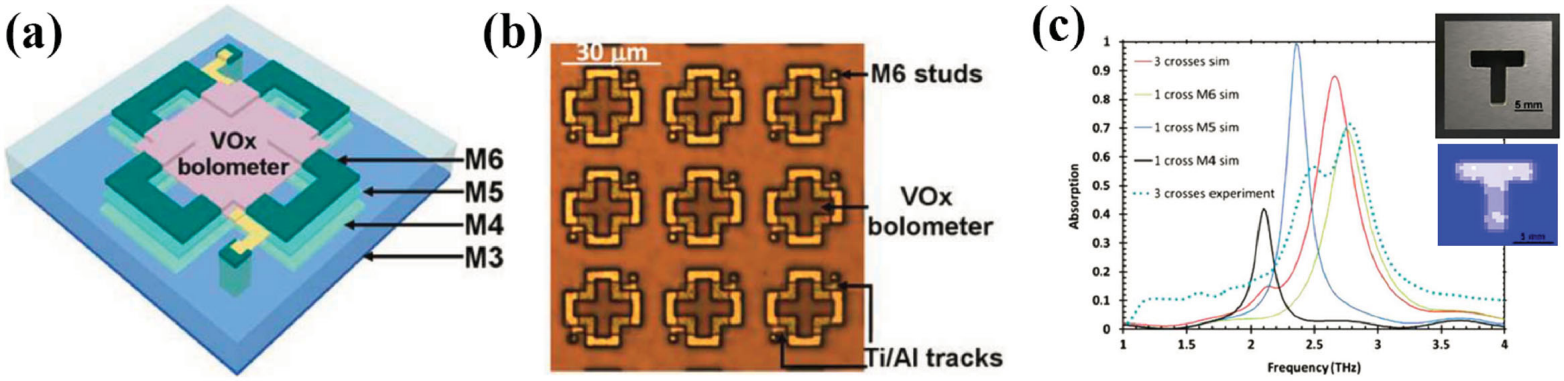
a) Schematic diagram of metamaterial‐based THz image ensor. b) Microscope image of a section of the post‐processed chip. c) Simulation and measured absorption spectral responses of metamaterials absorbers. Insets show the optical image of the aluminium cut out 'T' shape and its THz image taken at 2.5 THz using the metamaterial‐based THz image sensor. Reproduced with permission.^95^ Copyright 2015, IEEE.

THz imaging has been attracting research interests for the last one or two decades because THz radiation is non‐ionising, transparent to plastics and fibres, has higher resolution than millimeter wave, and matches in frequency with the characteristic absorption or vibration frequencies of some materials and biomolecules.^90^ Due to the weak interaction between THz radiation with most materials, most commercial THz detectors for imaging are typically comprised of discrete components that are bulky and expensive. Metamaterial with a ultrathin thickness has been investigated for absorption enhancement in visible, infrared and THz ranges.^91^, ^92^, ^93^ Cumming et al. proposed and demonstrated a monolithic resonant THz sensor element in 2013, where THz radiation was absorbed by metamaterial absorber and then detected by VO_2_ micro‐bolometer integrated to a CMOS chip.^94^ A minimum NEP of 37 pW Hz^–1/2^ and a thermal time constant of 68 ms was achieved. In 2015, they extended this single pixel sensor to a 5 × 5 pixel array with a pixel size of 30 μm × 30 μm.^95^ As shown in **Figure** **12**a, the metamaterial was made directly in the metallic and insulating layers available in the six metal layer CMOS foundry process. VO_2_ was deposited on top of Metal 6 and connected to the bottom electronics by post‐processing steps. The good quality of the processed pixel array and the expected absorption band centered at 2.5 THz can be seen in Figure 12b and 12c. The image of the aluminium cut out 'T' shape taken by this metamaterial‐based THz focal plane array shown in the inset of Figure 12c clearly demonstrated this functionality of this ultracompact THz sensor at room temperature.

### 4.4 Multispectral IS

Conventionally, color image sensors sample information through R, G, B channels and then show the named true color by signal processing. In this process, most spectral information is lost. In contrast, multispectral or hyperspectral imaging collects image data simultaneously in dozens or hundreds of narrow, adjacent spectral bands, which makes it possible to derive a continuous spectrum for each image cell for material diagnostic characterization.^96^ One of the most important techniques in multispectral IS is the method of dispersing the light spectrally. Compared to conventional dispersing elements such as a prism and a filter wheel that are bulky, expensive and not easy to use, a superpixel IS is preferable, where narrow‐band filters can be integrated on each cell of the superpixel. In this case, the detailed spectral information can be obtained from various cells of the superpixel. It is a unique property of nanophotonic color filtering techniques for tunable and fine spectral filtering beyond the broadband and material‐based filtering response of conventional dye doped polymers.

**Figure 13:**
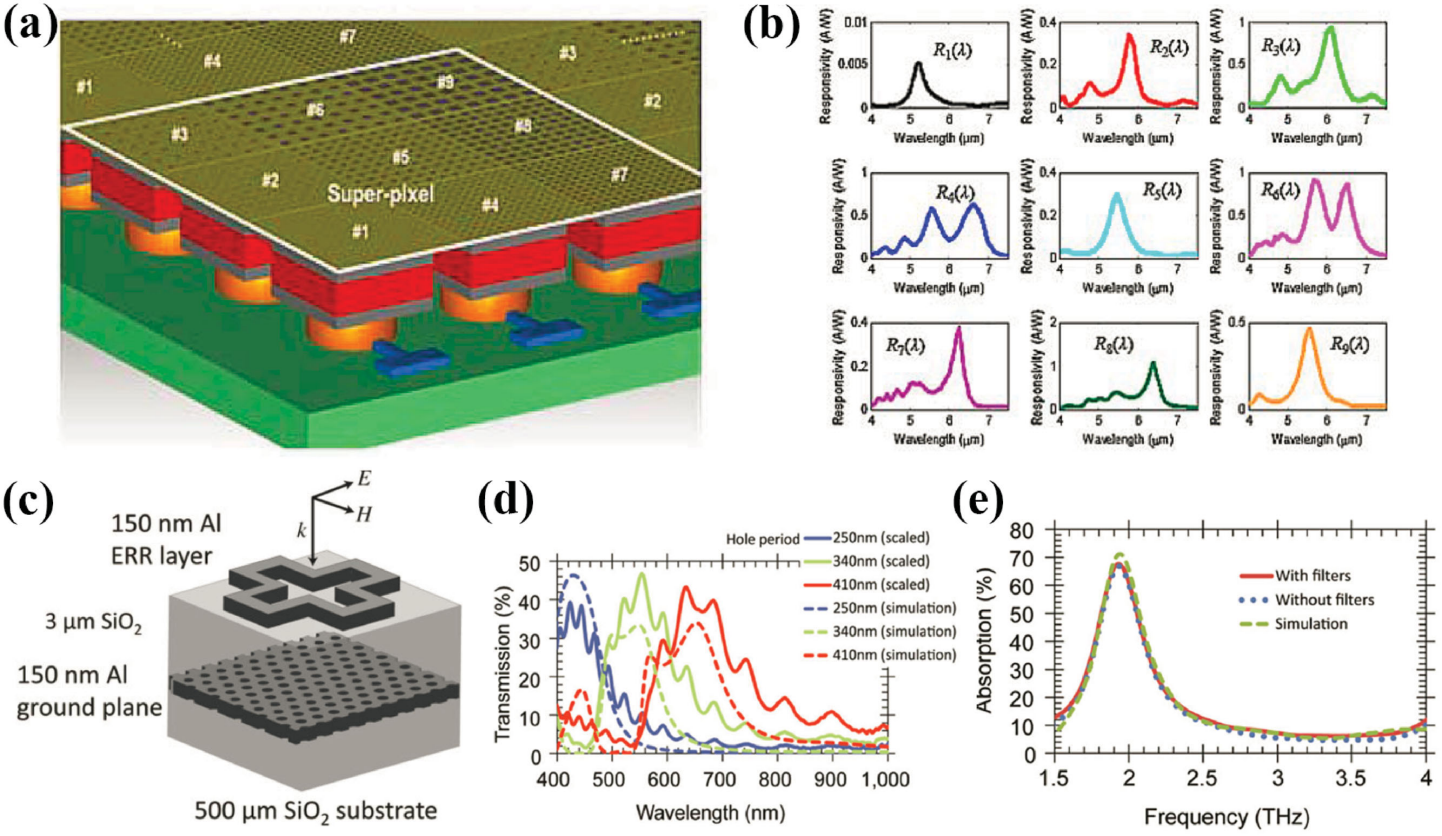
a) Schematic diagram of a plasmonic superpixel developed with nine different plasmonic filters integrated on top. b) Spectral responses of nine pixels in a superpixel. c) Schematic diagram of vertically cascaded THz and visible filters. d) Simulation and measured results of transmission spectra of the dual‐band filter in visible range. e) Simulation and measured absorption spectra of the dual‐band filter in THz range. a, b) Reproduced with permission.^100^ Copyright 2015, IEEE. c–e) Reproduced with permission.^103^

Various nanophotonic filter techniques have been investigated for multispectral imaging such as nanohole array, nanowire and multilayer film.^59^, ^97^, ^98^ However, the spatial resolution of image sensor decreases due to the superpixel configuration, where several tens bands, i.e. subpixels, are usually required to obtain detailed spectral information.^99^ Recently, algorithm‐coupled superpixel sensors were proposed incorporating spectra response modification through the use of plasmonic nanohole array filters.^100^, ^101^ As shown in **Figure** **13**a, nine different nanohole arrays were integrated in a superpixel. To extract multispectral information, the deconvolution process is applied to every superpixel after the algorithm processing. The spectral data at each pixel is an estimate of the spectral intensity. In this case, the sensor is capable of enhancing signals at resonant wavelengths, capturing and processing only the relevant information for reconstructing the spectrum. The choice of nanohole array filters is strictly made by considering the function of the compressive spectral sensing algorithm.^102^ Rather than the narrow‐band filtering responses, randomly distributed spectral responses were used to effectively sample the spectral information, which significantly compress the required number of individual filters, i.e. improving the spatial resolution.

Instead of multispectral imaging in a single band, Cumming et al. proposed a method for multi‐band spectral characterization.^103^, ^104^, ^105^ Figure 13c shows a unit cell of a visible and THz dual‐band filter, where a cross ring shape and a nanohole array were vertically cascaded. The nanohole array acts as color filters in visible range as discussed in Section 2. The electric ring resonance of the cross ring locates in the THz range and is applied for THz spectral characterization. Because the size of the cross ring is quite large compared to the visible wavelength and the filling ratio of the cross ring is small, the cross ring only introduces slight shielding effect for the underneath visible nanohole array color filters. In addition, because the large difference in wavelength for visible and THz waves, the nanohole array in aluminium film acts similarly as a uniform metallic reflector to THz wave. As a result, the stack consisting of Al cross ring layer, SiO_2_ and Al nanohole array forms a THz metamaterial absorber as the one in Figure 12 for THz filtering. Based on this vertically cascaded configuration, a dual‐band camera can be achieved by composing nanohole array filters with underneath silicon photodiodes (Figure 7) and THz metamaterial absorber based microbolometer (Figure 12). This excellent spectral manipulating capability of nanostructures rather than typical materials such as dye significantly improves the imaging capability.

## 5 Discussion

In SPP7 – the Seventh International Conference on Surface Plasmon Polaritons in 2015, nanophotonics community has a common point of view that structural color may be the second commercialized nanophotonic technique following plasmonic biosensing.^6^ The development of spectral filtering and light manipulating techniques in the last several years may continue the Moore's Law projection for image sensors; the smaller the better.^5^ For practical application of nanophotonic IS, some issues in existing techniques have to be addressed.

Although various nanophotonic techniques for spectral filtering have been proposed and demonstrated, even integrated into IS, no single technique has all the attributes of high light efficiency, high spectral purity, angular independence and process compatibility for the applications in IS. MIM filters are very simple and do not need high accuracy patterning techniques. But the spectral response is mainly determined by the thickness of the dielectric layer. Multistep lithography and high‐accuracy alignment are necessary for patterning R, G, B filters, which increases the cost and reduces the yield rate. This issue is more serious in the case of high resolution IS with a sub‐μm pixel or multispectral IS. Nanohole array filters with different spectral response could be fabricated simultaneously and have robust angular stability. But the transmittance is usually limited by the metal loss and the color purity is low. GMR filters have high transmittance and narrow passband, i.e. high light efficiency and high color purity. But it is very sensitive to the incident angle. It is difficult to predict which technique will be commercialized and be adopted in the next generation IS. The intrinsic advantages of GMR filters in light efficiency and color purity may eventually make it to be a good choice if the angular sensitivity is suppressed. In addition, an in‐depth study of crosstalk and size effect is desired considering the practical application in IS. So far, only few work has some preliminary investigations.^72^, ^80^ Currently, nanophotonic color filters still cannot overcome the conventional dye filters for the application of regular RGB color filtering. Compared to the conventional dye filters, the most unique property of nanophotonic spectral filters is the spectral tunability that is indispensable to multispectral imaging.^100^ The advantage to fabricate dozens of different filters in a single step is very attractive for superpixel multispectral IS. So does the multi‐band IS.^103^ These kinds of applications may be the first success of nanophotonic IS.

For nanophotonic elements integration to IS, there have been several methods, such as physical attachment,^72^ post‐CMOS process,^73^, ^74^, ^75^ back‐end‐of‐line CMOS process.^76^, ^77^, ^78^, ^88^, ^89^, ^94^, ^95^ Results of both photocurrent measurement and real imaging process prove the functions. Although the former two are achievable in laboratory research, the later one is a practical way. However, the CMOS foundry has fixed process parameters, for example metal layer thickness and positions. The nanophotonic optical elements have to be optimized to fit for this process rules at a cost of performance, unless the academia's research outcome convince the industry to modify their processes. Fortunately, several industry players including CEA LETI, ST Microelectronics, Panasonic, Sony, Samsung and LG have been involved in nanophotonic spectral devices and nanophotonic IS.^19^, ^76^, ^77^, ^78^ Considering the ultrasmall physical size of nanophotonic elements and their unique structure based properties, nanophotonic IS has a promising potential for the next generation IS with the development from both academia and industry.
